# Novel gene (TMEM230) linked to Parkinson’s disease

**DOI:** 10.1186/s40734-016-0046-7

**Published:** 2016-11-15

**Authors:** Diana A. Olszewska, Conor Fearon, Tim Lynch

**Affiliations:** 1 Department of Neurology, Dublin Neurological Institute at the Mater Misericordiae University Hospital, Dublin, Ireland; 2 Department of Neurology, Cork University Hospital, Cork, Ireland

**Keywords:** Tmem230, Transmembrane protein 230 gene, Parkinson’s disease, Genetics, Therapeutics

## Abstract

Mutations in six genes are known to cause Parkinson’s disease (PD) (autosomal dominant: alpha-synuclein, LRRK2, VPS35 and autosomal recessive: Parkin, PINK1 and DJ1) and number of other genes are implicated. In a recent article Deng and colleagues studied a large four generation American family of European descent and linked mutations in a novel gene, transmembrane-protein 230 gene (TMEM230) with lewy body confirmed PD. The authors demonstrated that pathogenic TMEM230 variants in primary mouse neurons affected movement of synaptic vesicles suggesting that TMEM230 may slow vesicular transport. Further experiments in HEK293 cells (carrying the pathogenic TMEM230 variants) showed increased alpha-synuclein levels. This study indicated that the impaired vesicular trafficking may contribute to the pathogenesis of PD. Understanding the various cellular mechanisms leading to PD may lead to the development of novel, much needed therapeutic options. These mechanisms could include: enhanced clearance of damaged mitochondria, development of kinase inhibitors, VPS35/retromer function enhancers or now the possibility of vesicular transport modification.

## Background

The pathogenesis of Parkinson’s disease (PD) involves the interaction of environmental and genetic factors. Although mutations in known PD genes are responsible for only 10 % of PD cases, the discovery of causative genes has improved our understanding of this incurable disease. Mutations in six genes are known to be causative of PD (autosomal dominant: alpha-synuclein-SNCA, LRRK2, VPS35 and autosomal recessive: Parkin, PINK1, DJ1) and number of other genes are implicated (EIF4G1, DNAJC13). The diagnosis of PD can only be confirmed pathologically by the loss of dopaminergic neurons in the substantia nigra pars compacta accompanied by the presence of Lewy bodies (LB) and Lewy neurites. Of note LB are absent in Parkin-associated PD [1].

### Novel gene TMEM230 linked with Parkinson’s disease in a large family study

Deng and colleagues, [2] recently, linked mutations in a novel gene, trans-membrane protein 230 gene (TMEM230), with LB-confirmed PD. The authors studied a large, four generation American family of mixed European descent (Dutch/German/Russian) with autosomal dominant PD. They used genome-wide linkage analysis in 13 affected family members to localise the candidate region at the short arm of chromosome 20 (10.7 Mb with 141 known genes). Subsequently, samples from four distant affected relatives and one healthy family member (age 87) were selected for whole-exome sequencing. Following the exclusion of variants with an average heterozygosity of more than 0.01 and filtering for variants resulting in functional change, a missense variant in TMEM230 (c.422G > T) was identified in four affected only. Co-segregation with the disease was confirmed by Sanger sequencing in other affected family members only (DNA analysis was performed in 65 family members including 13 with PD). This new gene variant was not present in the dbSNP database, 1000 Genomes Project database, Exome Sequencing Project database or 1238 controls. Individuals with c.422G > T variant had PD symptoms, a good levodopa response and mean age-at-onset 67 (range: 48–85). Two further non-significant variants in intronic regions were detected.

### Neuropathological examination

The neuropathological examination in three affected PD patients carrying TMEM230 c.422G > T variant confirmed the presence of LB and Lewy neurities in midbrain and neocortex.

### New variants in TMEM230

Further sequencing of 832 North American PD samples found two variants: c.275A-G in a 34-year-old man with PD and his unaffected mother (age 57) and c.551A-G in a 33- year-old affected man with a family history of PD in his maternal male cousin (disease onset at age 35 with no further information available). The pathogenicity of these two variants remains elusive. Sequencing of 574 Chinese PD samples led to the detection of a new c.550_552delTAGinsCCCGGG variant in 7 probands with familial PD, confirmed by a segregation analysis in two families. This variant was found in 7 (31 %) of 225 familial PD, but was not found in 10000 Chinese controls.

### Previous reports on the family

This is not the first time this Dutch/German/Russian family has been studied. Villarino-Guell and colleagues [1] previously reported a novel missense variant in DNAJC13 gene in the same family; however it did not fully co-segregate with the disease. The variant was absent in three PD patients and present in a healthy 87 year-old. Villarino-Guell et al. postulated that indeed the DNAJC13 variant may not be the sole cause of the disease [1].

### TMEM230 involvement in vesicular transport

Deng et al. [2] very nicely demonstrated that all four described pathogenic variants in primary mouse neurons adversely affected movement of synaptic vesicles suggesting that TMEM230 mutations slow vesicular trafficking. Co-localization with SNCA was also observed. Impaired vesicular transport and neurotransmitter release could lead to a failure of SNCA degradation and accummulation of SNCA in cells. Experiments in HEK293 cells carrying these variants demonstrated an increase in SNCA levels compared with controls. Impaired trafficking and recycling of vesicles could be a new causative mechanism in the pathogenesis of PD.

### Cellular mechanisms in Parkinson’s disease and their role in therapeutics

Understanding the various cellular mechanisms leading to PD may lead to the development of novel, much needed therapeutic options. These mechanisms could include: enhanced clearance of damaged mitochondria, development of kinase inhibitors, VPS35/retromer function enhancers or now the possibility of vesicular transport modification. It is well recognized that mutations in PINK1 and Parkin cause defects in mitochondrial homeostasis and accumulation of damaged mitochondria lead to neurodegeneration [3]. Parkin has housekeeping duties and development of Parkin enhancers leading to increased clearance of damaged mitochondria may have an important role in therapeutics [3]. Protein and lipid kinases are crucial in metabolism, cell signaling, and protein regulation. G2019S is the most common mutation in Leucine-rich repeat kinase 2 (LRRK2) and it may cause kinase activity up-regulation, proinflammatory response enhancement, and neuron toxicity [4, 5]. It was recently demonstrated that a novel kinase inhibitor (PF-06447475) attenuated α-synuclein-induced neurodegeneration in wild-type rats providing another hopeful therapeutic mechanism [4, 5]. Another PD gene VPS35 is a central scaffold for binding of other subunits of the retromer. The retromer is crucial in mediating retrograde transport of specific membrane proteins from endosomes back to the trans-Golgi network (hydrolases and proteases) and to the cell surface [6]. The retromer has an important role in secretion of signaling proteins (Wnt) and apoptotic cell clearance [6]. Absence of retromer activity causes decreased Wnt secretion and dopaminergic neuronal loss. Enhancement of intracellular transport occurs with an increase in the VPS35 level leading to modulation of retromer function [6]. As the enhancement of the retromer function reverses the neurotoxic effects without any obvious toxicity, the development of VPS35/retromer enhancers could be another therapeutic possibility [6]. VPS35 is linked to the trans Golgi network and possibly has a common pathway with newly described TMEM230; however, this has to be yet determined.

## Conclusions

The interplay between the gene products is important and the novel mechanism mediated by TMEM230 adds to current knowledge and may lead to targeting the modification of a vesicle trafficking as a novel therapeutic option.

## Abbreviations

DJ1: Parkinsonism Associated Deglycase
DNAJC13: DnaJ Heat Shock Protein Family (Hsp40) Member C13
EIF4G1: Eukaryotic translation initiation factor 4E
HEK293: Human Embryonic Kidney 293 cells
LB: Lewy body
LRRK2: Leucine-rich repeat kinase 2
PINK1: Phosphatase and tensin homolog-induced putative kinase 1
PD: Parkinson’s disease
SNCA: Alpha-synuclein
TMEM230: Transmembrane-protein 230 gene
VPS35: Vacuolar protein sorting-associated protein 35
